# 

**Published:** 1854-07

**Authors:** 


VOL. III. NEW SERIES. No. 7.

### THE NORTH-WESTERN

### MEDICAL AND SURGICAL

### JOURNAL:

EDITED BY

N. S. DAVIS, M.D.,

PROFESSOR OF PATHOLOGY, PRACTICE OF MEDICINE AND CLINICAL MEDICINE.

AND

H. A. JOHNSON, A.M., M.D.

LECTURER ON PHYSIOLOGY AND HISTOLOGY IN RUSH MEDICAL COLLEGE.

PUBLISHED MONTHLY,

AT TWO DOLLARS PER ANNUM IN ADVANCE.

JULY, 1854.

CHICAGO:

J. F. BALLANTYNE, PUBLISHER AND PRINTER,

NEW POST-OFFICE BUILDINGS, COR. OF CLARK AND RANDOLPH-STS.,
OPPOSITE THE SHERMAN HOUSE.

1854.

VOL XI. WHOLE SERIES. No. 7.

